# Routine feedback of test results to participants in clinic- and survey-based surveillance of HIV

**DOI:** 10.2471/BLT.15.153031

**Published:** 2015-03-16

**Authors:** Rachel Baggaley, Cheryl Johnson, Jesus Maria Garcia Calleja, Keith Sabin, Carla Obermeyer, Miriam Taegtmeyer, Basia Zaba, Carol El-Hayek, Jerome Amir Singh

**Affiliations:** a World Health Organization, avenue Appia 20, 1211 Geneva 27, Switzerland.; b Joint United Nations Programme on HIV/AIDS, Geneva, Switzerland.; c American University of Beirut, Beirut, Lebanon.; d Liverpool School of Tropical Medicine, Liverpool, England.; e London School of Hygiene & Tropical Medicine, London, England.; f Burnet Institute, Melbourne, Australia.; g CAPRISA, University of KwaZulu-Natal, Durban, South Africa.

## Abstract

Surveillance for human immunodeficiency virus (HIV) in low- and middle-income countries started in the 1980s. However, the questions of whether the results of HIV tests should be given to participants, and if so how, has still not been resolved. In the absence of effective treatment, it was considered acceptable to withhold results from HIV-positive participants. However, when antiretroviral treatment is available, some argue for beneficence – that it is the researcher’s duty to return the test results to all those who provide samples for surveillance. The corollary is that only participants who wish to receive their test results would be eligible to participate in surveys. Others argue for autonomy – that to obtain a more representative result for the general population, surveys should not exclude participants who do not wish to receive their test results. This round table discussion takes a closer look at those two arguments. We believe that the global community should work towards routine feedback of HIV surveillance while ensuring that participants receive and understand their test results.

## Résumé

La surveillance du virus de l'immunodéficience humaine (VIH) dans les pays à revenu faible et intermédiaire a commencé dans les années 1980. Les résultats des tests VIH doivent-ils être communiqués aux participants et si oui, comment ? En l'absence de traitement efficace, il avait été jugé acceptable de ne pas divulguer leur séropositivité aux participants infectés par le VIH. En revanche, dès lors qu'un traitement antirétroviral est disponible, certains évoquent le principe de bienfaisance et estiment qu'il est du devoir du chercheur de communiquer les résultats des tests à tous ceux qui ont fourni des échantillons à des fins de surveillance. Mais le corollaire est que seuls les participants qui acceptent d'être informés des résultats de leur test seraient alors éligibles pour participer à ce type d'enquêtes. D'autres avancent le principe d'autonomie, en disant que pour obtenir un résultat plus représentatif de la population générale, ces enquêtes ne doivent pas exclure les participants qui ne souhaitent pas recevoir leurs résultats. Cette table ronde examine en détail ces deux positions. Nous pensons que la communauté internationale devrait œuvrer pour une rétroaction systématique autour de la surveillance du VIH, en veillant à ce que les participants reçoivent et comprennent les résultats de leurs tests.

## Resumen

El seguimiento del virus de la inmunodeficiencia humana (VIH) en países de ingresos bajos y medios empezó en los años ochenta. ¿Deberían proporcionarse los resultados de las pruebas del VIH a los participantes? Si es así, ¿de qué modo? A falta de tratamiento efectivo, se consideró aceptable retener los resultados de los participantes VIH-positivos. Sin embargo, cuando se dispone de tratamiento antirretroviral, hay quien defiende la beneficencia, es decir, que es el deber del investigador informar de los resultados de las pruebas a todos aquellos que proporcionen muestras para el seguimiento. La consecuencia de esto es que solo los participantes que quieran recibir los resultados de sus pruebas reunirían los requisitos para participar en las encuestas. Otros defienden la autonomía, es decir, que para obtener un resultado más representativo de la población en general, las encuestas no deberían excluir a los participantes que no quieran recibir los resultados de sus pruebas. Este debate de mesa redonda analiza con más detenimiento estos dos argumentos. Creemos que la comunidad mundial debería trabajar por la información rutinaria del seguimiento del VIH, al mismo tiempo que garantiza que los participantes reciban y entiendan los resultados de sus pruebas.

## ملخص

بدأ الترصُّد لفيروس عوز المناعة البشري ( **HIV ** ) في البلدان المنخفضة الدخل والمتوسطة الدخل في ثمانينيات القرن العشرين. هل يجب إعطاء نتائج اختبارات فيروس عوز المناعة البشري إلى المشاركين، وإذا كان الأمر كذلك، فكيف؟ في ظل غياب المعالجة الفعَّالة، كان من المقبول حجب النتائج عن المشاركين الإيجابيين لفيروس عوز المناعة البشري. ولكن مع توافر المعالجة بمضادات الفيروسات القهقرية، يجادل البعض استناداً إلى فكرة الشفقة – بأنه من واجب الباحث أن يعيد نتائج الاختبار لجميع من قدموا العينات للترصُّد. فتكون النتيجة الطبيعية هي أن المشاركين الذي يريدون تلقي نتائج اختباراتهم هم فقط من يمكنهم المشاركة في المسوحات. بينما يجادل البعض الآخر استناداً إلى فكرة الاستقلالية –  بأنه حتى يمكن الحصول على نتيجة ممثلة لعموم السكان بصورة أكبر، فيجب ألا تقوم المسوحات باستثناء المشاركين الذين لا يريدون تلقي نتائج اختباراتهم. تلقي مناقشة المائدة المستديرة هذه نظرة فاحصة على هاتين الحجتين. نحن نؤمن بأنه يجب على المجتمع الدولي العمل على تقديم ملاحظات روتينية لترصُّد فيروس عوز المناعة البشري، وفي نفس الوقت ضمان تلقي المشاركين نتائج اختباراتهم واستيعابها.

## 摘要

在中低收入国家进行人体免疫缺损病毒 (HIV) 监测于 20 世纪 80 年代开始启动。是否应该将 HIV 检测结果告知受试者？如果是的话，应该采用何种方式？在缺乏有效治疗的情况下，人们认为向 HIV 阳性受试者隐瞒结果是可以接受的。但是，如今，随着抗逆转录病毒治疗的出现，有些人主张善行——认为研究人员有义务将测试结果告知所有提供样本用于监测的受试者。其必然结果是，只有希望获得测试结果的受试者才有资格参与抽样调查。有些人主张自主权——也就是为了获得能够用于大众的更具代表性的结果，抽样调查不应将不希望获得测试结果的受试者排除在外。本次圆桌会议进一步探讨了这两种观点。我们认为全世界都应该在确保受试者获得并理解其测试结果的同时，努力向 HIV 监测的常规反馈迈进。

## Резюме

Надзор за вирусом иммунодефицита человека (ВИЧ) в странах с низким и средним уровнем доходов начался в 1980-х годах. Следует ли отдавать результаты анализов на ВИЧ участникам и, если да, каким образом? В случае отсутствия эффективного лечения не сообщать результаты ВИЧ-позитивным участникам считалось приемлемым. Однако, если доступна антиретровирусная терапия, некоторые медицинские работники утверждают, что с точки зрения принципа «делай благо» исследователь обязан вернуть результаты анализа всем участникам, которые предоставили образцы для наблюдения. Таким образом, только участники, желающие получить результаты своих анализов, будут иметь право принимать участие в исследованиях. Другие медицинские работники являются сторонниками автономии — для получения более репрезентативного результата для населения в целом из исследования не стоит исключать участников, которые не хотят получать результаты своих анализов. Во время заседания за круглым столом мы обсудим эти два утверждения более подробно. Мы считаем, что мировое сообщество должно стремиться к тому, чтобы регулярно оповещать участников исследований о состоянии эпиднадзора за ВИЧ, информировать их о результатах анализов и пояснять эти результаты.

## Introduction

In the early stages of the human immunodeficiency virus (HIV) epidemic, surveillance presented various logistical and ethical issues – including whether or not the results of HIV tests should be given to all of the tested participants.^1^ There is a public health benefit if individuals are told that they have tested HIV-positive and then adopt preventive behaviours which limit further transmission. However, the absence of treatment at that time often created the perception that there were few personal advantages for HIV-positive individuals to receive their test results. These personal advantages were weighed against the potential risks individuals might experience, including social isolation, rejection and anxiety. There were concerns that, if HIV surveillance system data were insufficiently protected, disclosure could lead to further stigma and discrimination against HIV-infected individuals.^2^ In many settings, there were also resource concerns. If HIV surveillance systems required individual informed consent and receipt of test results, this might overburden surveillance staff.^3^ Before 2002, because of these perceptions and issues, much clinic-based HIV surveillance employed unlinked anonymous testing of remnant specimens – e.g. from syphilis testing. In such surveillance systems, HIV test results could not be returned to participants. Most protocols for population-based surveys of HIV prevalence included obtaining informed consent for testing but did not require disclosure of test results to all the participants.^3^

Today, the environment is different. Antiretroviral treatment is available in most settings. The availability of treatment has transformed the outlook of people with HIV infection and changed the perceptions of those conducting HIV surveillance. Antiretroviral treatment can, in addition, prevent both vertical (mother-to-child) and sexual transmission^2^^,^^4^ and is therefore of benefit to the uninfected population as well as people living with HIV.

In the 1990s, many countries with low-level, concentrated epidemics of HIV discontinued unlinked anonymous testing in HIV surveillance in favour of testing with informed consent and result disclosure. However the practice of unlinked anonymous testing has continued, particularly in antenatal clinics in countries in sub-Saharan Africa.^5^^,^^6^ Today, worldwide, in most clinic-based or population-based surveys of HIV, explicit consent is sought from participants to provide a sample for HIV testing. The results may be linked to behavioural and other personal data. In some cases, HIV surveys are conducted on the basis that participants may consent to provide a sample and may also decide whether or not to receive their result. However, this approach conflicts with standard practice in surveys of diabetes, hypertension, tuberculosis and many other treatable conditions, in which individuals consent to participate on the basis that they will always receive their test results.^7^

## Global debate

In 2013, the Joint United Nations Programme on HIV/AIDS (UNAIDS) and the World Health Organization (WHO) issued guidance on how to assess the availability and quality of data collected as part of a programme for the prevention of mother-to-child transmission of HIV.^8^ It is anticipated that, in the near future, enough data of high quality will be available from such programmes that there will no longer be any need for unlinked anonymous testing in antenatal clinics. However, more detailed guidance is still needed for countries that decide to use programmatic data for HIV surveillance.

In September 2014, WHO and UNAIDS hosted a global meeting to update their *Guidelines for using HIV testing technologies in surveillance*.^9^ Country representatives, laboratory specialists, surveillance experts and programmatic experts who participated in this meeting debated the issue of returning the results of HIV tests, collected during surveys, to the tested individuals. They reviewed published arguments for surveillance methods in which all tested participants are given their test results^10^^,^^11^ and compared them with those in which test results would only be provided if requested.^12^^,^^13^ The discussions considered different types of surveillance – e.g. national population-based cross-sectional surveys and community-based longitudinal surveys. The different ethical issues relating to individuals known to be HIV-positive, those who had not previously been tested and those who had previously tested HIV-negative but were still exposed to risk of infection were also discussed. Since point-of-care testing for HIV surveillance should not replace diagnostic testing, the need for all test results to be confirmed according to national testing algorithms was also highlighted.

In general, the meeting participants tended to adopt two different positions: those who believed that there should be automatic individual feedback of results and those who believed that survey participants should be able to opt out of knowing their test results. Both viewpoints are grounded in the principles of biomedical ethics, with the former placing emphasis on beneficence and the latter on autonomy.

### Argument for feedback

Those who argued for automatic feedback said that any other approach would be unethical, given the unmet demand for HIV testing, the wide availability of treatment and the potential benefit to participants of knowing their test results. As part of their informed consent, potential survey participants should be asked to provide demographic and other relevant information. They should also be offered a chance to provide a test sample, in the knowledge that, if tested, they will always be told the test result. However, no samples should be collected from participants who declared that they did not want to know their HIV status. Although some people may decline to participate in HIV surveillance if they know they will automatically receive their test results, most individuals offered HIV testing in clinical^14^ or community-based settings^15^ have agreed to be tested. Furthermore, it would be consistent with clinic-based and population-based surveillance conducted for other treatable conditions, where those who are tested are automatically informed of their test results and referred for care.

### Argument against feedback

The alternative argument was that, although survey participants should be encouraged to receive their test results, surveys should not require participants who provide samples to be informed of their test results as a condition of participation. Such an approach, which should promote participation and reduce survey participation bias, would allow each potential participant to make two discrete choices: (i) whether to participate in the surveillance; and (ii) for those who agree to participate, whether to receive their test result. Proponents of this view point out that people who already know that they are HIV-positive – so-called known positives – and people who arrange to be frequently retested – so-called repeat testers – may agree to participate and be tested but choose to decline to receive their results.

Some of those who argue against the automatic feedback of test results concede that such feedback may be justified when there is likely to be just one opportunity for a participant to be told their test result. The women included in HIV surveillance done in clinics for antenatal care or the prevention of mother-to-child transmission, for example, may not receive HIV testing again or be seen by those operating HIV surveillance. In multi-round longitudinal surveys and community-based research surveys, however, there may be multiple opportunities for participants to receive their test results. In these contexts, known positives and repeat testers are often encountered within a well-defined population that is surveyed at regular intervals.

Despite these differences in the opinions of the meeting participants, supporters of the automatic provision of test results maintain that information and counselling provided for repeat testers and known positives can be reduced and tailored to these groups – just as in the clinical settings where HIV testing is routinely offered and known positives and repeat testers may also be encountered.

## Different values and views

Those who argue that all individuals tested for HIV should automatically be told their HIV status tend to believe that autonomy should chair, not rule.^16^ That is, respect for participants’ autonomy should not over-rule the ethical principle of beneficence. Since surveys usually have eligibility criteria, survey participants do not have any particular right to take part in a survey. Surveys that are not based on consent to the automatic individual feedback of test results are perceived as being untenable – because of the participants who remain unaware that they have been found positive in an HIV test and because of the interviewers who have not passed on test results to people that they have found to be HIV-positive.

Among those who argue against such automatic feedback, there is a belief that – to increase methodological rigour and obtain results that may be more representative of the general population – HIV surveys should be conducted in a way that does not exclude participants who do not wish to receive their test results. The use of protocols that require participants to receive their test results tends to reduce the participation of individuals who know they are living with HIV.^17^^,^^18^ For those who argue against automatic feedback, the public health value of better knowledge about the HIV epidemic outweighs any disadvantage associated with not providing test results.

## Towards routine feedback

The consensus view that came out of the September 2014 meeting was that the global health community should be working towards ensuring that individuals who participate in HIV surveillance studies routinely receive their HIV test results. There remain concerns about the accuracy of HIV test results in the context of surveillance and, particularly, whether such results should be communicated to participants as definitive diagnoses or initial indicators of HIV status that needed further confirmation. Every HIV test result should be communicated to the tested individual in a way that enables the individual to understand the meaning of the result, respond to the result appropriately and, importantly, obtain relevant test confirmation, prevention, care, support and treatment services.

Programmes for HIV surveillance must consider how they can best ensure that participants receive and understand their test results.
